# A Case of Partial Inversion of the Uterus

**Published:** 1878-10

**Authors:** Henry T. Byford


*A Case of Partial Inversion of the Uterus:*

Mrs. F—, German, aged 42 years, large, muscular and generally healthy, and mother of five children, was delivered of the last on the 16th of January, 1878, by a midwife. The labor was attended by no unusual circumstance, and the placenta removed without pain or delay. After delivery there was an unusually large amount of blood lost, but not sufficient to alarm either the midwife or the patient, who, although weak, was considered to be in a fair post-partum condition. She continued to have profuse sero-sanguineous discharges, became exhausted, and, in consequence, very much alarmed.

This imperfect history is all I could glean from those surrounding the patient at the time I was first called to see her, viz : the 24th of January, eight days after confinement. She was nearly bloodless, and presented all the symptoms of acute anæmia, such as languor, listlessness, despondency, tinnitus, palpitation, œdema, great pallor, rapidity and weakness of pulse, inability to move without a sense of faintness, etc. The appetite was gone, the tongue coated, the bowels were constipated, and there was an entire inability to swallow or retain any solid food. As her stomach was so weak she had not attempted to take any but fluid nourishment, hence her diet since confinement had consisted almost entirely of fennel and beef teas. The discharge from the vagina still continued, but was not very profuse.

Although I suspected some grave accident had happened to the uterus, in the presence of such an untoward condition of the patient, I determined to delay a thorough vaginal examination until I could induce a more vigorous state of the circulation. With this view I prescribed the tincture of iron and sul. quinia, internally; ordered perfect quietude, poached eggs and milk, as much as the stomach would bear, and, as soon as that organ became a little more tolerant, the tender portions of young pigeons. This course was continued perseveringly, the food being tolerated well, until January 28th, when she was very decidedly improved, although, during the few days previous, the discharge had become a little more profuse. Her pulse had gained vigor and become slower; the countenance was more cheerful, the lips more natural in color, and motion much better tolerated; she had now become hopeful, and was beginning to take an interest in her household affairs. At this visit I ventured to make a vaginal examination. I found the os uteri patulous, dilated to about the size of a silver dollar, and filled with the fundus which extended slightly below the level of the external border of the firm ring in which it was grasped. By means of one hand placed above the symphysis, while two fingers of the other were in the vagina, a cup shaped depression, showing the absence of fundus uteri, was easily recognized. There was evidently a partial inversion of the uterus present; the cervical portion of the organ was firm while the fundus and body, as well as I could determine by palpation, were soft and impressible. Reflecting on this state of things, and believing my patient in too prostrated a condition for the manipulation necessary to restore the fundus, I was led to hope also, that if a little tone could be imparted to the fibers of the body and fundus, the discharge would diminish and possibly a spontaneous restoration result. Continuing the former supporting treatment and quietude, I administered half a drachm of Squibb's fluid extract ergot every four hours. From the time the ergot was commenced the discharge gradually became less, the fundus and body firmer, and the os smaller until the 28th of February, 1878, when the uterus was restored to its proper size, shape and position. The os was closed, the sound entered the uterine cavity three inches, and penetrated three inches into the uterine cavity by deep pressure above the symphysis, while two fingers pushed the uterus upward, the natural outlines of the fundus could be distinguished. Tonics and a nutritious diet were continued from this time forward, and were followed by uninterrupted improvement, until the patient was entirely cured.

My father, Dr. Wm. H. Byford, saw the patient with me February 10th. He fully verified my diagnosis, and coincided with me in the treatment. He also examined the patient August 16th, 1878, and assured me that the uterus was not only restored to its natural position, but was in a healthy condition.

I will not presume to make any comments on this case, as the points will readily present themselves to the reader. The only thing to which I will draw attention is the steady improvement following the administration of ergot. It is true, nature might have done all that was necessary for the restoration of the uterus without this remedy; it certainly produced no injurious effects.

In this connection it may be apropos to mention a case which was under the observation of my father where spontaneous restoration took place after the uterus had been completely inverted for fifteen years.

Henry T. Byford, M. D.

